# Mental Health Status and Coping among Portuguese Higher Education Students in the Early Phase of the COVID-19 Pandemic

**DOI:** 10.3390/ejihpe13020032

**Published:** 2023-02-10

**Authors:** Carlos Laranjeira, Maria Anjos Dixe, Ana Querido

**Affiliations:** 1 School of Health Sciences of Polytechnic of Leiria, Campus 2, Morro do Lena, Alto do Vieiro, Apartado 4137, 2411-901 Leiria, Portugal; 2 Centre for Innovative Care and Health Technology (ciTechCare), Rua de Santo André-66-68, Campus 5, Polytechnic of Leiria, 2410-541 Leiria, Portugal; 3 Comprehensive Health Research Centre (CHRC), University of Évora, 7000-801 Évora, Portugal; 4 Center for Health Technology and Services Research (CINTESIS), Innovation & Development in Nursing, University of Porto, 4200-450 Porto, Portugal

**Keywords:** mental health, higher education students, stress, anxiety, depression, coping, COVID-19, pandemic, Portugal

## Abstract

Globally, the COVID-19 outbreak had an adverse effect on higher education students’ mental health and psychological well-being. This study aims to assess the prevalence of stress, anxiety, depression and associated factors in a sample of students in the early phase of the COVID-19 pandemic and determine the predictive effect of mental health status on coping. The sample was collected between March and July 2020 and included 392 higher education students in Portugal. An online cross-sectional study was conducted using a survey that included an information form, the Depression, Anxiety, and Stress Scale, and the Brief Resilient Coping Scale. The prevalence of mild-to-extremely severe depression, anxiety and stress was 24.2%, 32.7% and 33.4%, respectively. About 60% of the sample had poor coping abilities. Masters students, participants older than 30 years and female participants had significantly greater resilient coping compared to undergraduate students and younger and male participants (*p* < 0.05). Resilient coping correlated negatively with depression, anxiety and stress. The regression analysis showed that age together with overall levels of depression, anxiety and stress explained 16.9% of the variance in coping. The results should inform the implementation of interventions to mitigate the impact of psychological distress and promote mental health.

## 1. Introduction

The first case of infection by the new coronavirus was detected in late 2019, and the virus is considered the biggest public health emergency facing the world in recent decades [1]. With the rapid spread of COVID-19 worldwide, some countries adopted contingency measures in order to reduce the number of infections and deaths, namely measures focused on social isolation [2,3]. Therefore, in addition to physical health risks and concerns, the pandemic also implied a set of negative consequences for the mental health of the entire population [1,3,4,5].

Student mental health has long been a major concern. Pre-pandemic research on student health revealed high rates of mental health problems, with a pooled prevalence of depression symptoms ranging between 27% and 34% [6,7,8]. Students are sensitive to psychopathological symptoms because of factors such as curriculum structure, the pressure to achieve academic standards, frequent assessments, high functional stress, fear of failure, a competitive learning environment and a lack of time for self-care and interaction with family and friends [9]. While leaving the parental home is frequently associated with feelings of increased independence and autonomy as well as a reduction in parental control, it can also result in homesickness, difficulties adjusting to one’s new environment, isolation and feelings of inadequacy [10,11].

Besides the fact that changing environments and lifestyles are quite often stressful for students starting university, pandemics have certainly exacerbated this problem, as documented by recent reviews of the psychological impacts of COVID-19 on college students [1,12,13]. Isolation, loneliness and concern about catching the disease were found to be drivers of poor psychological health [14,15,16]. The uncertainty in university education (including concerns about online courses and evaluations) and sudden changes in life (e.g., social isolation, lower family income and fewer job prospects) led to increased feelings of stress, anxiety and depression among many students [17]. Furthermore, according to some studies, quarantine conditions in particular have generated or worsened psychopathological symptoms with possible short- and long-term consequences on the mental health of individuals [2]. Research has identified symptoms of depression, anxiety, stress [18,19,20,21], symptoms of post-traumatic stress, confusion and anger associated with the pandemic situation [22]. Some studies reported suicide cases that seem related to the psychological impact of COVID-19 [23,24]. These impacts were observed in universities worldwide [25].

Resilience and coping can act as protective factors, mitigating negative psychological impacts such as trauma, threats and high stress levels [26]. However, resilience and coping are conceptually different concepts. In positive psychology, resilience is considered a dynamic, complex and multifaceted construct. Individual variations in the ability to reduce distress and foster adaptation were observed throughout the COVID-19 pandemic [27]. Previous data suggest that psychological resilience is negatively correlated with symptoms of depression and anxiety, which underlines its protective effect regarding physical and mental status [28,29]. In contrast, coping is conceptualized as dealing with stress and adversity in a positive and highly adaptive manner [30,31]. Evidence suggests that people with greater resilient coping were less likely to exhibit emotional problems [32].

To date, few studies have explored COVID-19′s impact on the mental health of university students in Portugal, specifically psychopathological symptoms (e.g., depression, anxiety and stress). Previous studies reported a high prevalence of depression, anxiety and stress in this population [33,34], but they did not measure how participants coped. Therefore, this study aimed: (a) to assess the prevalence of stress, anxiety and depression levels as well as resilient coping in a sample of Portuguese higher education students; (b) to investigate the influence of student characteristics (age, sex, marital status, type of school, level of studies and field of study) on resilient coping; (c) to evaluate the degree of association between mental health status and resilient coping; (d) to determine the predictive effect of mental health status (depression, anxiety and stress levels) on the resilient coping of students.

More specifically, we hypothesized that: (H1) the prevalence of mental health problems (depression, anxiety and stress levels) among Portuguese higher education students is high, and levels of resilient coping are low; (H2) there are significant differences in resilient coping scores based on student characteristics; (H3) there is a strong association between mental health status and resilient coping; (H4) depression, anxiety and stress are strong predictors of resilient coping.

## 2. Materials and Methods

### 2.1. Study Design

This study used cross-sectional data collected between April and July of 2020 and targeted higher education students (aged ≥18 years) during a lockdown period in Portugal due to the spread of the COVID-19 pandemic.

### 2.2. Recruitment and Sample

The sample was drawn from four public higher-education institutions (i.e., universities and polytechnic institutes) and covered several education programs. The targeted institutions, located in the central region of Portugal, are medium-sized with similar activity profiles. The sample size was calculated using a single population proportion formula (Raosoft sample size calculator) [35], assuming an estimated population of 40,000 students (an average of ten thousand students per institution). According to this calculation, our sample size should contain at least 381 students (for an estimated 50% response distribution, 95% confidence level and a margin of error ±5%). Sample calculation was used to avoid type I and type II errors.

A convenience sampling method was applied. Due to the imposed lockdown, the required data were collected digitally with a link to the study questionnaire (Google Form) and distributed via institutional email and relevant academic e-platforms. IP filtering was employed to prevent repeated responses and duplicates from one system, reducing the risk of selection bias.

All participants were given information to help them make an educated decision about whether to participate in the study. They were given explanations of the scales and the study’s goal. Participants who accepted to take part in the study completed an informed consent form by checking the “Yes, I Agree” box on the online form rather than the “No thanks” box. Participation was voluntary and unpaid.

### 2.3. Instruments

The online survey included three domains of questions/measures: (a) demographic information; (b) the Depression, Anxiety and Stress Scale (DASS-21); (c) the Brief Resilient Coping Scale (BRCS).

(a) Demographic items included age, sex, marital status, type of school, level of studies and study area.
(b) The Portuguese version of the Depression, Anxiety and Stress Scale (DASS-21) [36], originally developed by Lovibond and Lovibond [37], was used to assess mental health status. Each of the 21 items (seven per subscale) was assessed on a four-point Likert scale ranging from “never” (0) to “always” (3), with higher scores indicating higher symptom levels. Based on percentiles matching Lovibond and Lovibond’s cut-offs, the prevalence in each subscale was separated into five levels. Thus, “the total depression subscale score was divided into normal (0–9), mild depression (10–12), moderate depression (13–20), severe depression (21–27), and extremely severe depression (28–42); the total anxiety subscale score was divided into normal (0–6), mild anxiety (7–9), moderate anxiety (10–14), severe anxiety (15–19), and extremely severe anxiety (20–42); and the total stress subscale score was divided into normal (0–10), mild stress (11–18), moderate stress (19–26), severe stress (27–34), and extremely severe stress (35–42)” [38] (p. 14). In this study, Cronbach’s alpha was 0.92, 0.89 and 0.92 for the Depression, Anxiety and Stress subscales, respectively.
(c) The Portuguese version of the Brief Resilient Coping Scale (BRCS) was used to assess an individual’s tendency to cope adaptively [39]. This unidimensional scale was originally created by Sinclair and Wallston [30] and includes four items: “1. I look for creative ways to alter difficult situations; 2. Regardless of what happens to me, I believe I can control my reaction to it; 3. I believe that I can grow in positive ways by dealing with difficult situations; 4. I actively look for ways to replace the losses I encounter in life” [30] (p. 98). Every item has a score range from 1 (Does not describe me at all) to 5 (Describes me very well), where higher scores indicate higher resiliency. Resilience was classified into three categories according to the following cut-off points: “low resilience (4–13), medium resilience (14–16), and high resilience (17–20)” [17] (p. 329). Cronbach’s alpha of the scale for this study was 0.83.

### 2.4. Data Analysis

The sample was characterized with descriptive statistics (e.g., mean, standard deviation, frequencies and percentages). Differences in resilient coping scores between students with different demographic characteristics were assessed using the independent-samples *t*-test and one-way analysis of variance (ANOVA) followed by the Bonferroni post hoc test. A *p*-value < 0.05 indicated a statistically significant result in both the *t*-test and the ANOVA.

Bivariate associations between the subscales of DASS-21 and resilient coping were estimated using Pearson’s correlation coefficient. Additionally, a stepwise logistic regression was conducted to identify the significant predictors of resilient coping. We classified respondents into medium/high resilient coping (BRCS score ≥ 14) and low resilient coping (BRCS score ≤ 13). Age, sex and level of studies were inserted as covariates in all four steps. The sequential entry of predictors was drawn based on the strength of the bivariate correlation between mental health symptoms and resilient coping (depression was the first variable to enter the regression model, followed by stress and anxiety). The low intraclass correlation (ICC of 0.00–0.02) when considering the type of institution and field of studies suggests these variables are not relevant for understanding differences in resilient coping. Hence, these variables were not entered into the logistic regression analysis. Variables with a *p*-value > 0.05 were excluded from the regression analyses. All statistical tests were conducted using the Statistical Package for Social Sciences (SPSS version 27 for Windows, IBM Corp., Chicago, IL, USA).

## 3. Results

Three hundred and ninety-two Portuguese higher education students between 18 and 58 years of age participated in this study (mean = 25.02; SD = 8.501). Table 1 provides further detail on the sample’s characteristics. Overall, 81% of participants identified as female and 19% as male. Most of the students were single (85%) undergraduates (83%) from polytechnic institutions (65%) and studying Healthcare Sciences (49%); the remaining students were studying Economics (13%), Technologies (12.2%), Law (9.7%), Architecture (5.6%) or other fields (10.5%).

Descriptive statistics related to depression, anxiety, stress and coping resilience are also given in Table 1. The overall prevalence of mild-to-extremely severe depression, anxiety and stress were 24.2%, 32.7% and 33.4%, respectively. The BRSCS score indicated that 236 (60.2%) students exhibited low levels of resilient coping, whereas 89 (22.7%) showed moderate levels and 67 (17.09%) showed high levels.

Comparing resilient coping levels according to the demographic and academic characteristics of the students, a significant difference in resilient coping scores was found between sexes (*t*  =  0.188, *p* < 0.05), with female students scoring better than male students. Resilient coping and age were also significantly correlated (*F* = 4.416, *p* < 0.05). Post hoc tests indicated that students 30 years and older exhibited higher resilient coping scores compared to other students ((18–20); (20–30), *p* < 0.05). Furthermore, there was a significant association between stress and the level of studies (*F* = 2.016, *p* < 0.05). Post hoc tests showed that postgraduate students had higher resilient coping scores than undergraduate students (*p* < 0.05). There were no significant differences in resilient coping levels among students based on their marital status, type of school and field of study.

To assess the levels of association among depression, anxiety, stress and resilient coping, a correlation matrix was calculated. Significant correlations were found between all variables (Table 2). The DASS-21 depression scale (r = −0.348, *p* < 0.01), the DASS-21 anxiety scale (r = −0.177, *p* < 0.01) and the DASS-21 stress scale (r = −0.247, *p* < 0.01) were all negatively associated with the resilient coping instrument (BRCS). Similarly, depression, anxiety and stress scales were positively correlated with each other.

For the purpose of the current study, we performed a hierarchical linear regression analysis to assess the effects of overall levels of depression, anxiety and stress on resilient coping. The demographic variables “Age”, “Sex” and “Level of studies” were added in the first block (Model I); “Depression” was added in the second block (Model II); “Stress” was added in the third block (Model III); “Anxiety” was added in the fourth block (Model IV). The first model only explained 3.1% of the variance in resilient coping, whereas the second model explained an additional 9% of the variance; the third model explained an additional 2%; the fourth explained an additional 2.8% of the variance in coping. All models were significant. Furthermore, as shown in Table 3, age (*β* = 0.136; *p* < 0.05), depression (*β* = −0.327; *p* < 0.001), stress (*β* = −0.178; *p* < 0.05) and anxiety (*β* = −0.064; *p* < 0.05) were strong predictors of resilient coping (R^2^ = 0.169).

## 4. Discussion

This study examined depression, anxiety, stress and resilient coping in a sample of Portuguese higher education students facing the initial phase of the COVID-19 pandemic. Although there is already literature on the consequences of pandemics on mental health, this topic is recent and current, and therefore, additional research on the topic is relevant, especially related to mental health, a topic often cast aside [40].

The study participants had levels of depression, anxiety and stress similar to those reported in studies with university students in the early stages of the pandemic [21,41]. However, compared to previous Portuguese COVID-19 investigations, our results show somewhat lower levels of depression, anxiety and stress [38].

Maia and Dias [34] compared results from 2018/2019 with those obtained during the onset of the pandemic and identified a significant increase in the levels of anxiety, depression and stress among university students. Our results revealed a lower prevalence of anxiety and stress and a higher prevalence of depression than those found in a recent global synthesizing study, wherein the prevalence of depression, anxiety and stress were 37%, 29% and 23%, respectively [42]. Thus, the COVID-19 pandemic had effects on the mental health of university students [21,34,43]. Our initial lack of information about the virus and the pandemic’s unpredictability contributed towards a stressful experience, possibly resulting in acute and uncontrolled emotional responses that manifested as anxiety and depressive symptoms [44].

The present study found significant effects of gender, age and level of studies on resilient coping, even though there was no consistency across previous studies [45,46].

Some “studies conducted on resilience, coping, and COVID-19 reveal sex-related differences” [46] (p. 4), with female students revealing lower resilience levels than male students [47]. In contrast, in our study, female students scored better than male students. We speculate that women, generally more empathic than men, perceive social support more intensely, which improves their resilient coping [47]. In previous outbreaks, harsher psychological impacts were associated with younger and less educated people [48], which is in line with our findings.

Correlational analysis showed significant negative correlations between coping levels and depressive/anxious/stress symptoms, a well-established relationship in previous studies related to the COVID-19 pandemic [49,50]. The lockdown rules restricting students to their homes may have constrained their capacity to solve problems and develop coping strategies [44]. Stress is undoubtedly a part of student life and may affect the ability to cope with the demands of university life [18]. Differences in symptom severity may be explained by differences in coping responses and the ability to interpret contextual cues. Therefore, preventative interventions based on these resilience components can help reduce psychopathological symptoms among students [51]. Furthermore, the present study verified the significant influence of mental health levels on resilient coping. In most previous studies, mental health status appeared only as an outcome measure, with few reports studying positive mental health as a predictive factor [52]. Our study contributes to this type of analysis, indicating that improved mental health is related to increased resilient coping. Indeed, these results can be useful for developing psychological interventions aimed at students, following the pandemic. Possible mitigation strategies include providing positive pandemic-related information, learning to manage stress, improving family relationships, promoting positive behaviours and adjusting academic expectations [53,54]. Individual or group resilience training programs that combine diverse formats (e.g., face-to-face and telephone coaching) could be also beneficial. This sort of intervention, based on the problem-solving model of stress, may promote psychological adaptability to stress, particularly by strengthening the resilience element of active coping [55]. In addition, faculty educators should consciously build resilience-fostering and empowering learning environments using student-centred pedagogies without compromising academic rigour [56]. Examples of such strategies include reducing cognitive load by strengthening soft skills and interpersonal competencies, rethinking evaluations and adopting trauma-informed education [57].

Considering the global impact of COVID-19 worldwide, fostering psychological well-being has become critical [4,58,59]. Our study provides a basis for a model that incorporates the variables that influence mental health status and resilient coping during the COVID-19 crisis. Future research should incorporate intervening factors into a structural equation model to validate the current assumption.

There are certain limitations to this exploratory study that should be addressed in future research and should be considered when interpreting the findings. First, due to the sampling procedure and non-probabilistic strategies used, the sample lacks statistical representativeness, which might have resulted in selection bias. Similarly, voluntary participation via an internet platform might generate bias due to motivation or lack of technological access to the data-collecting form. Second, the cross-sectional design does not identify causal links between variables. Our data was collected two months after the pandemic began; thus, we cannot report on changes over time or, more importantly, whether the elevated levels of stress, anxiety and depression were transient or long-term. We were also unable to differentiate between pre-existing cases versus new cases of depression, anxiety and stress. In addition, “the DASS-21 offers only a snapshot of recent mental health symptomology”, and the students “may not wish to disclose mental health symptoms in an attempt to avoid stigma and biases associated with mental health disorders” [60] (p. 11). Therefore, we may have been unable to identify participants with high scores of depression and stress levels on the DASS-21. Nonetheless, our results reveal high psychological distress in the early phase of the COVID-19 pandemic in Portugal. A prospective and longitudinal investigation might provide insights into the causes of psychological distress during the pandemic. Lastly, the self-assessment measures for depression, anxiety, stress and resilient coping, while reliable and used generally, do entail reporting and social desirability bias, which can affect outcomes.

## 5. Conclusions

During the ongoing COVID-19 crisis, there were drastic changes in behaviour, social thinking and individual daily routines because of the preventive measures (e.g., social distancing) necessary to reduce the risk of contagion. In addition to the impact on physical health, these changes affected the financial, social and mental health of individuals all over the world. In this survey, mild-to-extreme symptoms of stress were more prevalent than depression and anxiety complaints. Our results are aligned with available evidence that indicates that the pandemic aggravated the levels of anxiety, depression and stress of university students, a population that is at risk for the development of mental disorders, even in non-pandemic situations. In this sense, understanding how to deal with the changes and consequences caused by COVID-19 is important, as different coping strategies can lead to different impacts, positive or negative, on the well-being and health of the individual.

## Figures and Tables

**Table 1:** Sample’s characteristics (n = 392).

Variables		n	%
Age M ± DP = 25.02 ± 8.501	(18–20)	67	17.1
	(20–30)	242	61.7
	>30	83	21.2
Sex	Female	316	80.61
	Male	76	19.39
Marital status	Single	332	84.69
	Married	53	13.52
	Divorced	6	1.53
	Widowed	1	0.26
Type of school	Polytechnic institution	253	64.5
	University institution	139	35.5
Level of studies	Undergraduate level	326	83.16
	Post-graduate level	8	2.04
	Master’s level	42	10.71
	Other	16	4.08
Field of study	Natural Sciences	17	4.34
	Healthcare Sciences	192	48.98
	Technologies	48	12.24
	Architecture, Fine Arts and Design	22	5.61
	Education Sciences and Teacher Training	13	3.32
	Law, Social Sciences and Services	38	9.69
	Economics, Management and Accounting	51	13.01
	Humanities, Secretariat and Translation	6	1.53
	Physical Education, Sport and Performing Arts	5	1.28
DASS-21 depression scale	Normal (≤9)	297	75.77
	Mild depression (10–12)	37	9.44
	Moderate depression (13–20)	54	13.78
	Severe depression (21–27)	4	1.02
	Extremely severe depression (≥28)	0	0.00
DASS-21 anxiety scale	Normal (≤6)	264	67.35
	Mild anxiety (7–9)	48	12.24
	Moderate anxiety (10–14)	51	13.01
	Severe anxiety (15–19)	22	5.61
	Extremely severe anxiety (≥20)	7	1.79
DASS-21 stress scale	Normal (≤10)	261	66.58
	Mild stress (11–18)	108	27.55
	Moderate stress (19–26)	23	5.87
	Severe stress (27–34)	0	0.00
	Extremely severe stress (≥35)	0	0.00
Resilient coping scale	Low resilience (4–13)	236	60.20
	Medium resilience (14–16)	89	22.70
	High resilience (17–20)	67	17.09

**Table 2:** Descriptive and correlational analysis between depression, anxiety, stress and resilient coping.

	(Min–Max)	Mean	SD	1	2	3	4
1. DASS-21 depression	(0–42)	12.81	10.976	-			
2. DASS-21 anxiety	(0–42)	10.36	10.415	0.707 **	-		
3. DASS-21 stress	(0–42)	17.02	11.258	0.772 **	0.796 **	-	
4. Resilient coping	(4–20)	12.82	3.553	−0.348 **	−0.177 **	−0.247 **	-

Min, minimum value; Max, maximum value; SD, standard deviation; ** *p* < 0.01.

**Table 3:** Hierarchical multiple regression analysis predicting resilient coping in higher education students (n = 392).

	Model I			Model II			Model III			Model IV		
	B	SE B	*β*	B	SE B	*β*	B	SE B	*β*	B	SE B	*β*
Age	0.074	0.021	0.177 ***	0.041	0.020	0.098 *	0.045	0.020	0.107 *	0.125	0.036	0.136 *
Sex	−0.036	−0.037	0.008	0.005	−0.005	0.077	−0.120	−0.100	0.060	−0.132	−0.109	0.033
Level of studies	0.044	0.043	0.006	0.077	−0.013	0.058	0.021	0.039	0.016	0.28	0.011	0.50
DASS-21 depression	-			−0.113	0.015	−0.348 ***	−0.138	0.022	−0.427 ***	−0.156	0.122	−0.327 ***
DASS-21 stress	-			-			0.051	0.023	−0.149 *	0.089	0.146	−0.178 *
DASS-21 anxiety	-			-			-			0.053	0.524	−0.064 *
R^2^	0.031			0.121			0.141			0.169		
F	12.654 ***			21.028 ***			29.019 ***			53.580 ***		

B—unstandardized beta; SE B—standard error for the unstandardized beta; *β*—standardized beta * *p* < 0.05; *** *p* < 0.001.

## Data Availability

All data generated or analysed during this study are included in this article.
